# Tomato juice intake increases resting energy expenditure and improves hypertriglyceridemia in middle-aged women: an open-label, single-arm study

**DOI:** 10.1186/s12937-015-0021-4

**Published:** 2015-04-08

**Authors:** Asuka Hirose, Masakazu Terauchi, Moe Tamura, Mihoko Akiyoshi, Yoko Owa, Kiyoko Kato, Toshiro Kubota

**Affiliations:** 1 Department of Obstetrics and Gynecology, Tokyo Medical and Dental University, Yushima 1-5-45, Bunkyo, Tokyo 113-8510 Japan; 2 Department of Women’s Health, Tokyo Medical and Dental University, Yushima 1-5-45, Bunkyo, Tokyo 113-8510 Japan

**Keywords:** Menopausal symptoms, Anxiety, Basal metabolism, Dyslipidemia

## Abstract

**Background:**

Tomato-based food products have health-promoting and disease-preventing effects. Some tomato juice ingredients may have health benefits for middle-aged women, including women with menopausal symptoms and cardiovascular diseases. We investigated the net effect of tomato juice intake on several health parameters in women in this age group.

**Methods:**

An open-label, single-arm study was conducted, involving 95 women (40-60-years-old) who had at least one menopausal symptom. The participants refrained from foods and drinks rich in tomato and tomato-based products for 2 weeks prior to the study and during the 8 weeks of tomato juice consumption. After the run-in period, the women were asked to consume 200 mL of unsalted tomato juice, twice daily for 8 weeks. Their menopausal symptoms were evaluated using the Menopausal Symptom Scale (MSS), Hospital Anxiety and Depression Scale (HADS), and Athens Insomnia Scale (AIS) before the study, and at 4 and 8 weeks after study commencement. At the same times, body composition; blood pressure; heart rate; resting energy expenditures (REEs); and serum levels of triglyceride (TG), cholesterol, glucose, and hemoglobin A1c were measured.

**Results:**

Ninety-three women (98%) completed the study. The following parameters showed significant changes, compared with baseline, at study weeks 4 and 8 (mean ± standard deviation at baseline, week 4, and week 8): (1) the MSS score improved (9.9 ± 5.2, 8.5 ± 5.0, 8.3 ± 5.0; P < 0.0001, repeated measures analysis of variance(ANOVA)), (2) the HADS-anxiety subscale score improved (5.3 ± 2.7, 4.8 ± 2.4, 4.9 ± 2.9; P = 0.041, Friedman test), (3) heart rate increased (62.6 ± 9.4 bpm, 64.4 ± 8.6 bpm, 63.8 ± 8.2 bpm; P = 0.028, Friedman test), (4) REE increased (1980 ± 368 kcal/day, 2108 ± 440 kcal/day, 2149 ± 470 kcal/day; P = 0.0030, repeated measures ANOVA), (5) serum TG level decreased in the subgroup of women (n = 22) who had high TG (150 mg/dL or higher) at baseline (237.8 ± 88.9 mg/dL, 166.7 ± 86.1 mg/dL, 170.9 ± 109.7 mg/dL; P = 0.0002, Friedman test).

**Conclusions:**

Tomato juice intake alleviated menopausal symptoms, including anxiety, increased REEs and heart rate, and lowered high baseline serum TG levels in middle-aged women.

**Trial registration:**

UMIN-CTRUMIN000011877.

## Background

Middle-aged women are not only bothered by the physical and psychological symptoms of menopause, but are also at increased risk for cardiovascular diseases (CVDs), such as central obesity, hypertension, dyslipidemia, and diabetes [1-4], which are partly induced as a result of diminished estrogen production [5]. CVD is the number one cause of death worldwide, accounting for 17.3 million (30%) deaths, globally, in 2008 [6]. Most CVDs could be theoretically prevented through an elimination of risk factors, such as tobacco use, unhealthy diets, obesity, high blood pressure, impaired glucose tolerance, and raised lipids [7]; however, modification of one’s own lifestyle is often difficult.

Tomato juice contains a variety of bioactive ingredients, such as gamma-aminobutyric acid (GABA), lycopene, 13-oxo-9,11-octadecadienoic acid (13-oxo-ODA), and esculeoside A, which may provide physical and psychological health benefits for middle-aged women. For example, GABA reduces psychological stress [8] and lowers blood pressure [9]. Lycopene has been reported to have anti-cancer effects and anti-oxidative effects, and to be effective at mitigating cardiovascular diseases, osteoporosis, and mental disorders [10-14]. Recently, 13-oxo-ODA was shown to lower plasma and hepatic triglyceride (TG) levels in an animal model of obesity [15]. Esculeoside A, a tomato saponin, was also shown to reduce serum levels of TG and cholesterol, and to ameliorate atherosclerotic lesions in ApoE-deficient mice [16]. Compared to fresh tomatoes, tomato juice has several advantages, including an increased level of extractable lycopene [17], and elevated antioxidant capacity (as a result of activation through the canning (heating) process) [18], and additionally, 13-oxo-ODA is found only in tomato juice [15]. Moreover, a recent research showed that tomato vinegar beverage improved glucose tolerance in high-fat diet-induced obese mice [19].

In the present study, we investigated the net effect of tomato juice intake on a variety of health parameters in middle-aged women, based on the hypothesis that anti-oxidative effect of lycopene and relaxing effect of GABA may alleviate physical and psychological symptoms of menopause; 13-oxo-ODA and Esculeoside A may increase resting energy expenditure (REE) and lower the serum level of triglyceride.

## Methods

We conducted an open-label, single-arm study at the Menopause Clinic of the Tokyo Medical and Dental University. The study protocol was reviewed and approved by the Tokyo Medical and Dental University Review Board, and written informed consent was obtained from all participants. The study was conducted in accordance with the Declaration of Helsinki [20].

Ninety-five Japanese women participated in this study. The inclusion criteria were as follows: ages between 40 and 60; having at least one menopausal symptom on the Menopausal symptom scale (MSS) (score >1). The exclusion criteria were as follows: medication for hypertension, dyslipidemia, diabetes, or other cardiovascular diseases; intake of vitamins, lycopene, GABA or other supplements that could affect the parameters to be evaluated; allergy to tomato or tomato products. The participants were recruited through advertisements posted in our hospital and in the patients’ social network. The participants were classified as follows: premenopausal (regular menstrual cycles in the past 3 months), perimenopausal (a menstrual period within the past 12 months but a missed period or irregular cycles in the past 3 months), postmenopausal (no menstrual period in the past 12 months), or had surgically or medically induced menopause (hysterectomy or chemotherapy for breast cancer).

From 2 weeks before the start until study termination, the participants refrained from foods and drinks rich in tomatoes and tomato products. After the run-in period, each participant was asked to consume 200 mL of unsalted tomato juice (Nippon Del Monte, Gunma, Japan) twice daily, just before breakfast and dinner, for 8 weeks. The nutritional composition of the tomato juice used in the current study is shown in Table 1. The product was manufactured in compliance with the Food Safety System Certification (FSSC) 22000 adopted by the Global Food Safety Initiative (GFSI). Adherence to the study protocol was confirmed by checking the participants’ diaries including the records of tomato juice consumption.

**Table 1:** **The nutritional composition of the tomato juice used in the current study**

Nutrient	Value per 400 ml
Energy	82 kilocalories
Protein	4.4 g
Fat	0 g
Sugars	14.4 g
Dietary fiber	3.6 g
Sodium	32 mg
Calcium	46 mg
Potassium	1260 mg
Vitamin A	92 μg
Lycopene	44 mg
13-oxo-ODA	78.4 μg
GABA	198 mg
Esculeoside A	unknown

13-oxo-ODA, 13-oxo-9,11-octadecadienoic acid.

GABA, gamma-aminobutyric acid.

The participants’ menopausal symptoms were evaluated using MSS, Hospital Anxiety and Depression Scale (HADS), and Athens Insomnia Scale (AIS) before, and after 4 and 8 weeks of study participation. At each time point, body composition, blood pressure, heart rate, and REE, as well as serum levels of TG, total cholesterol, low-density lipoprotein (LDL) cholesterol, high-density lipoprotein (HDL) cholesterol, and plasma levels of glucose and hemoglobin A1c (HbA1c) were measured.

The MSS was validated and used in previous studies for participants to rate the severity of ten menopausal symptoms [21]. We evaluated vasomotor symptoms (hot flashes, perspiration, and chilliness); somatic symptoms (irregular heartbeat, headache/dizziness, tiredness, and aching joints/muscles); and psychological symptoms (insomnia, irritability, and depressed mood) using a 4-point Likert scale, depending on how often each symptom affected their daily life: none (never, 0 points); mild (rarely, 1 point); moderate (sometimes, 2 points); severe (very often, 3 points). MSS scores were calculated as the total score for the 10 aforementioned symptoms.

Developed by Zigmond and Snaith as a questionnaire [22], the HADS is a reliable instrument for screening clinically significant anxiety and depression in women; the questionnaire was translated into Japanese by Kitamura et al. [23]. The AIS was developed as a brief, easy-to-administer self-assessment questionnaire for determining insomnia severity according to the International Classification of Disease, Tenth Revision. The internal consistency and test-retest reliability of the AIS was previously confirmed [24]. The current study followed the protocol reported earlier [25], who measured the effect of supplementation with grape seed proanthocyanidin extracts on determining insomnia severity using AIS [24] and screening of clinically significant anxiety and depression using the Japanese version of HADS questionnaire [23].

The body composition of the participants, including height, weight, body mass index, fat mass, and muscle mass, was assessed using a body composition analyzer (MC190-EM; Tanita, Tokyo, Japan). Participants’ systolic and diastolic blood pressure and heart rate were also measured using a vascular screening system (VS-1000; Fukuda Denshi, Tokyo, Japan). Additionally, REE was measured using a portable, indirect calorimeter (Metavine-N VMB-005 N; Vine, Tokyo, Japan).

Blood samples were collected by antecubital venipuncture and were centrifuged for the collection of serum. The serum and plasma samples were sent within 3 days of sampling to SRL, Inc. (Tokyo, Japan). The levels of serum lipids, plasma glucose, and HbA1c were assayed according to standard techniques.

Statistical analyses were performed using GraphPad Prism version 5.02 (GraphPad Software, San Diego, CA, USA) and IBM SPSS Statistics version 20 (IBM Corporation, Armonk, NY, USA). After testing the normality of each valuable using Shapiro-Wilk test, the variables with Gaussian distribution were evaluated with parametric tests (One-way repeated measures ANOVA and paired *t*-test) and those without were with non-parametric tests (Friedman and Wilcoxon signed-rank tests). One-way ANOVA and Kruskal-Wallis test were used for the evaluations of baseline characteristics among four different menopausal status groups and postprandial time for blood sampling. P-values < 0.05 were considered statistically significant.

## Results

Table 2 presents the characteristics at baseline and at 4 and 8 weeks after study commencement of the ninety-three women (98%), who completed the 10-week study. 41 women (44%) were premenopausal, whereas 17 (18%) were perimenopausal, 28 (30%) were postmenopausal, and 7 (8%) had surgically or medically induced menopause. Comparing the baseline data among the four different menopausal status groups, we observed no statistically significant differences except for serum levels of total cholesterol (premenopausal, 197.8 ± 31.2 mg/dL; perimenopausal, 231.9 ± 25.3 mg/dL; postmenopausal, 223.3 ± 31.1 mg/dL; had surgically or medically induced menopause, 216.3 ± 40.3 mg/dL; P = 0.0005, One-way ANOVA). The mean interval between the blood sampling time and the previous meal at the 0-, 4-, and 8-week measurements were: 3.8 ± 2.2 hours, 4.1 ± 2.6 hours, and 4.2 ± 2.5 hours, respectively (*P* = 0.62, Kruskal-Wallis test).

**Table 2:** **Participant characteristics at baseline and at 4 and 8 weeks after study commencement**

	Baseline	4 weeks	8 weeks	P-value
Menopausal symptom score				
MSS score	9.9 (5.2)	8.5 (5.0)**	8.3 (5.0)***	<0.0001^a^
HADS-anxiety subscale score	5.3 (2.7)	4.8 (2.4)^#^	4.9 (2.9)^#^	0.04^b^
HADS-depression subscale score	4.7 (3.2)	4.6 (3.4)	4.4 (3.3)	0.32^b^
Athens Insomnia Scale score	3.7 (2.3)	3.4 (2.4)	3.8 (2.8)	0.16^b^
Body composition				
Height, cm	157.5 (4.9)	157.5 (4.9)	157.5 (4.9)	0.50^b^
Weight, kg	55.0 (8.5)	54.9 (8.6)	55.0 (8.6)	0.13^a^
Body mass index, kg/m^2^	22.2 (3.2)	22.1 (3.2)	22.2 (3.2)	0.34^a^
Fat mass, kg	15.7 (6.1)	15.8 (6.2)	15.7 (6.2)	0.58^b^
Muscle mass, kg	37.0 (3.1)	36.9 (3.0)	37.1 (3.0)	0.046^a^
Blood pressure and heart rate				
Systolic blood pressure, mmHg	116.9 (14.1)	117.8 (14.3)	117.0 (13.8)	0.87^b^
Diastolic blood pressure, mmHg	75.4 (10.4)	75.2 (9.7)	74.5 (9.3)	0.23^b^
Heart rate, min^−1^	62.6 (9.4)	64.4 (8.6)^##^	63.8 (8.2)^##^	0.03^b^
Resting Energy Expenditure	1980 (368)	2108 (440)**	2149 (470)**	0.003^a^
Blood examination				
Triglycerides, mg/dL	115.9 (84.0)	116.3 (70.2)	107.4 (75.3)	0.25^b^
Total cholesterol, mg/dL	213.1 (33.4)	213.9 (36.1)	212.2 (36.8)	0.76^a^
LDL cholesterol, mg/dL	120.3 (28.8)	121.1 (31.2)	120.2 (32.2)	0.87^a^
HDL cholesterol, mg/dL	73.2 (16.3)	75.3 (14.9)	74.6 (15.0)	0.31^a^
Glucose, mg/dL	98.9 (16.6)	98.8 (11.6)	99.2 (19.0)	0.51^b^
HbA1c, %	5.3 (0.4)	5.3 (0.4)	5.4 (0.4)	0.36^b^

Data are shown as means (standard deviations).

^a^One-way repeated measures analysis of variance.

^b^Friedman test.

**P < 0.01, ***P < 0.001 versus baseline, Paired *t*-test.

^#^P < 0.05, ^##^P < 0.01 versus baseline, Wilcoxon signed-rank test.

We evaluated the changes in menopausal symptom severity using 3 scales. The mean MSS score (9.9 ± 5.2, at baseline; mean ± standard deviation) was significantly improved after 4 (8.5 ± 5.0, *P* = 0.0012, paired *t*-test) and 8 (8.3 ± 5.0, *P* < 0.0001) weeks of tomato juice consumption. The mean HADS-anxiety subscale score also improved, relative to baseline (5.3 ± 2.7) after 4 (4.8 ± 2.4, *P* = 0.0170, Wilcoxon signed rank test) and 8 (4.9 ± 2.9, *P* = 0.0337) weeks. There were no significant changes in the mean HADS-depression subscale and AIS scores after 4 and 8 weeks of the intervention.

We also evaluated the changes in participant body compositions, blood pressure, heart rate, and REEs. The participant body composition parameters and blood pressures did not change significantly after 4 and 8 weeks of intervention. However, the mean heart rate was significantly increased, compared to baseline (62.6 ± 9.4 bpm), after 4 (64.4 ± 8.6 bpm, *P* = 0.0046, Wilcoxon signed rank test) and 8 (63.8 ± 8.2 bpm, P = 0.0020) weeks of the intervention. In addition, the mean REE increased after 4 (2108 ± 440 kcal/day, *P* = 0.0082, paired *t*-test) and 8 (2149 ± 470 kcal/day, *P* = 0.0018) weeks of tomato juice consumption, relative to baseline (1980 ± 368 kcal/day).

**Figure 1:**
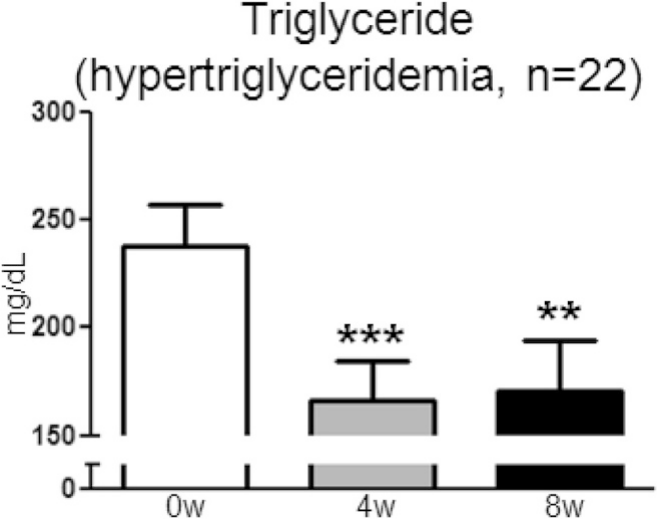
**Change in serum TG level in the subgroup of women who had hypertriglyceridemia at baseline (≥150 mg/dL).** Data are presented as means and standard errors. **P < 0.01, ***P < 0.001 versus baseline, Wilcoxon signed-rank test.

Finally, we evaluated the changes in serum levels of lipids and glucose. Although the serum TG level among all participants did not change significantly, in the subgroup of women (n = 22) with high TG levels at baseline (150 mg/dL or higher; actual, 237.8 ± 88.9 mg/dL), the level decreased significantly after 4 (166.7 ± 86.1 mg/dL, *P* = 0.0002, Wilcoxon signed rank test) and 8 (170.9 ± 109.7 mg/dL, *P* = 0.0025) weeks of intervention (Figure 1). The serum levels of total cholesterol, LDL cholesterol, HDL cholesterol, glucose, and HbA1c did not change significantly.

## Discussion

This open-label, single-arm study investigated the net effect of tomato juice intake on a variety of health parameters in middle-aged women. The results indicated that tomato juice intake alleviated some menopausal symptoms, including anxiety; increased REE and heart rate; and lowered serum TG levels (in women with elevated baseline levels).

Tomato juice contains a variety of ingredients, and the outcomes of this study should be summed effects of them. Most of the clinical studies on the effects of tomatoes, tomato juice, and tomato products have focused on lycopene, a bioactive ingredient that is abundant in tomatoes. Dietary intake of fresh tomatoes or tomato juice is known to increase the serum lycopene level [26]. Lycopene is expected to reduce cardiovascular diseases, osteoporosis, and mental disorders through its anti-oxidative effects [10-14]. In 2012, Kim et al. isolated 13-oxo-ODA from tomato juice. They showed that this juice component acted as a potent peroxisome proliferator-activated receptor-alpha (PPARα) activator to lower plasma and hepatic TG levels in an animal model of obesity [15]. Further, in 2010, Nohara et al. demonstrated that esculeoside A, a tomato saponin, reduced serum levels of TG and cholesterol, and ameliorated atherosclerotic lesions in ApoE-deficient mice [16]. Human studies have not been published, to our knowledge. We believe that the current study is the first to investigate the net effects of tomatoes or tomato products on the health parameters, including menopausal symptoms, body composition, blood pressure, heart rate, and serum levels of lipids and glucose, of middle-aged women.

In our study, menopausal symptoms improved following regular consumption of tomato juice over a prolonged (8-week) period. Although the mechanisms causing the vasomotor symptoms that affect middle-aged women are not yet fully understood, oxidative stress could be one of the responsible factors [27]. Psychological symptoms are likewise prevalent around menopause, and may be associated with oxidative stress [28]. The improvements in menopausal symptoms observed in our study participants could partly be explained by the anti-oxidative effect of lycopene. A meta-analysis of 13 clinical trials [12] showed that the least effective amount of lycopene to reduce oxidative stress was 10 mg/day, much lower than the estimated dosage in the current study, 44 mg/day. Furthermore, GABA, much more abundant in tomatoes than in other vegetables and fruits [29], could be another candidate as its analog, gabapentin, has been proven to relieve hot flush [30].

Our study also revealed that tomato juice intake increased REE and heart rate, and, in women with elevated baseline TG levels, lowered serum TG levels. 13-oxo-ODA is a newly identified PPARα activator, first reported in 2012 [15]. In their study, Kim et al. showed that mouse rectal temperatures were significantly higher in 13-oxo-ODA-treated animals than in the controls, implying that 13-oxo-ODA increased energy metabolism. Their study also showed that plasma, liver, and skeletal muscle TG levels were lower in the mice treated with 47.6 mg/kg of 13-oxo-ODA than controls. On the other hand, Nohara et al. focused on a tomato saponin, esculeoside A [16]. They showed that administration of 100 mg/kg/day of esculeoside A inhibited the accumulation of cholesterol esters in macrophages, and reduced serum levels of TG and cholesterol in ApoE-deficient mice. The increase in energy metabolism and the decrease in serum TG level shown in our study could be attributable to these bioactive ingredients, although caution should be taken as the estimated dosage of 13-oxo-ODA in the current study, ~1.4 μg/kg, is far smaller than that in the animal study, and we do not know the exact amount of esculeoside A contained in the tomato juice used. Further human studies investigating the effects of 13-oxo-ODA and esculeoside A on energy metabolism are warranted. The increase in heart rate observed in the current study may be associated with raised REE, as it is well known that heart rate is linearly related to basal energy expenditure [31].

The present study has some limitations. First, it is designed as a single-arm study because an adequate placebo or active control for tomato juice is difficult to prepare. Second, blood samples should rather have been collected in fasting status, which were unfortunately not acceptable for many participants in our study. However, the fact that the average postprandial intervals were not significantly different among the groups would justify the comparison between them. Third, some women may have changed their eating and exercise habits during the course of the 10-week study, although we requested them to adhere to their current lifestyles. Finally, we should rather have evaluated insulin resistance by measuring plasma insulin concentration instead of HbA1c in our relatively healthy participants.

## Conclusions

Tomato juice intake alleviated menopausal symptoms, including anxiety, increased REEs and heart rate, and lowered high baseline serum TG levels in middle-aged women.

## Abbreviations

MSS: Menopausal symptom scale
HADS: Hospital anxiety and depression scale
AIS: Athens insomnia scale
REE: Resting energy expenditure
TG: Triglyceride
ANOVA: Analysis of variance
CVD: Cardiovascular disease
GABA: Gamma-aminobutyric acid
13-oxo-ODA: 13-oxo-9,11-octadecadienoic acid
FSSC: Food Safety System Certification
GFSI: Global Food Safety Initiative
LDL: Low-density lipoprotein
HDL: High-density lipoprotein
PPARα: Peroxisome proliferation-activated receptor-alpha
